# *In Vitro* Global Gene Expression Analyses Support the Ethnopharmacological Use of *Achyranthes aspera*

**DOI:** 10.1155/2013/471739

**Published:** 2013-12-15

**Authors:** Pochi R. Subbarayan, Malancha Sarkar, Lubov Nathanson, Nikesh Doshi, Balakrishna L. Lokeshwar, Bach Ardalan

**Affiliations:** ^1^Division of Hematology and Oncology, Department of Medicine, University of Miami Miller School of Medicine, 1550 NW 10th Avenue, Fox 431A (D8-4), Miami, FL 33136, USA; ^2^Department of Biology, University of Miami, Miami, FL 33124, USA; ^3^Department of Medicine, University of Miami Miller School of Medicine, Miami, FL 33136, USA; ^4^Institute for Neuro Immune Medicine, College of Osteopathic Medicine, Nova Southeastern University, Fort Lauderdale, FL 33328, USA; ^5^Department of Urology, University of Miami Miller School of Medicine and VA Medical Center, Miami, FL 33136, USA

## Abstract

*Achyranthes aspera* (family *Amaranthaceae*) is known for its anticancer properties. We have systematically validated the *in vitro* and *in vivo* anticancer properties of this plant. However, we do not know its mode of action. Global gene expression analyses may help decipher its mode of action. In the absence of identified active molecules, we believe this is the best approach to discover the mode of action of natural products with known medicinal properties. We exposed human pancreatic cancer cell line MiaPaCa-2 (CRL-1420) to 34 **μ**g/mL of LE for 24, 48, and 72 hours. Gene expression analyses were performed using whole human genome microarrays (Agilent Technologies, USA). In our analyses, 82 (54/28) genes passed the quality control parameter, set at FDR ≤ 0.01 and FC of ≥±2. LE predominantly affected pathways of immune response, metabolism, development, gene expression regulation, cell adhesion, cystic fibrosis transmembrane conductance regulation (CFTR), and chemotaxis (MetaCore tool (Thomson Reuters, NY)). Disease biomarker enrichment analysis identified LE regulated genes involved in Vasculitis—inflammation of blood vessels. Arthritis and pancreatitis are two of many etiologies for vasculitis. The outcome of disease network analysis supports the medicinal use of *A. aspera*, viz, to stop bleeding, as a cure for pancreatic cancer, as an antiarthritic medication, and so forth.

## 1. Introduction

Large sections of the world population depend on medicinal plants (natural products) for their health care needs. The indigenous population use crude preparations of natural products in the form of decoction, paste, or as food supplement. The prevailing belief is that crude mixtures contain many compounds. Several of these compounds might be inactive by themselves but are essential for the effectiveness of the “active molecule” in the crude preparations. In other words, synergism negates the additive effect of individual molecules [1]. Global gene expression changes effected by nonstandardized (crude) preparations as used by communities at large may not be directly attributed solely to the “active molecule.” Understandably, this approach will not facilitate deciphering the mechanism of action of the active molecules in a crude preparation. However, global gene expression analyses will give a snapshot of the overall effect of crude extract on multiple cellular pathways [2]. Such approaches may explain the possible mode of action of herbal preparations as used in indigenous medicine.

Modern scientific standards emphasize thorough understanding of the molecular mechanism of action of single agent drugs. However, this approach is limited to single molecules, which have been chemically characterized. Even these drugs modulate the activities of disparate metabolic/signaling pathways [3]. These results suggest that a drug with specific target can have wide ranging physiological effects.

Analytical methods like microarray, pathway focused PCR arrays, RNA seq, and cGH array give a snapshot of large scale changes at genome level. These methodologies generate large data sets. The development of data mining software has enabled discovery of molecular markers from the mass of data possible. Thus, the ability to analyze gene expression changes at genome level had revolutionized the molecular investigations that until now were difficult. For example, understanding global gene expression changes that accompany certain diseases like cancer has enabled the development of personalized medicine. Data analysis of microarray results also facilitated deciphering molecular pathways that are affected by both existing as well as newly discovered drugs [2].

*Achyranthes aspera* (Family *Amaranthaceae*) is a medicinal plant in Ayurvedic pharmacopoeia. It is an annual shrub found in India, Southeast Asia, America, and Sub-Saharan Africa. It is classified as a weed [5]. Traditionally, the plant extract is used as a cure for arthritis, as blood clotting agent, to induce labor (fetal expulsion), and as an abortifacient [6]. Earlier, we demonstrated that methanol-extract of *A. aspera* leaves (LE) has antiproliferative activity against a variety of human cancer cell lines cultured *in vitro*. Our results also showed pancreatic cancer cells to be the most sensitive to LE. These exciting outcomes indicate the presence of active anticancer molecule(s) in *A. aspera*. Unpublished, anecdotal observations suggest that *A. aspera* benefited pancreatic cancer patients. Animal studies carried out with *A. aspera* extract confirm LE to be nontoxic to mice. Most importantly, these studies have shown that LE inhibited human tumor growth in mice [7]. In preliminary gene expression studies, we demonstrated LE differentially modulated the expression of key genes involved in vasculogenesis (VEGFs), metastasis (MMPs, TIMPS), apoptosis (Caspase), cell proliferation (Akt-1), and so forth [7, 8]. These preliminary results support the idea that LE regulates the function of specific signaling molecules. Except for the above two reports, no additional information is available regarding the global molecular changes caused by LE in human cancer cells. Clearly, simultaneous analysis of global gene expression changes effected by LE would shine light on different pathways affected. Such analyses would help select key genes that can be used as response markers and predict its effect on different illnesses. Based on this premise, we used Agilent Whole Human Genome microarrays to examine the global gene expression changes effected by LE on cultured human pancreatic cancer cell line MIA PaCa-2 (CRL-1420) that were treated with LE for 24, 48, and 72 hours. In this paper, we summarize the effect of methanol extract (LE) of dried powdered leaves of *A. aspera* on global gene expression pattern in human pancreatic cancer cell line MIA PaCa-2. Using MetaCore software, we narrowed down the pathways affected in MIA PaCa-2 cells treated with LE for three different time points.

## 2. Materials and Methods

We have recently published the details about the collection, authentication of *Achyranthes aspera* leaves, and the preparation of whole leaf extract [7, 8]. Briefly, *A. aspera* leaf powder was serially extracted in 100% hexane followed by 100% acetone over night at room temperature. The acetone extract was concentrated in a rotary evaporator. The residue was dissolved in 100% methanol and called leaf extract (LE).

### 2.1. Cell Lines and Culture Conditions

The human pancreatic cancer cell line MIA PaCa-2 (CRL-1420) was purchased from ATCC (USA) and maintained in our laboratory in RPMI 1640 medium supplemented with 10% FBS (Invitrogen, USA) at 37°C in a humidified 5% CO_2_ atmosphere. It was seeded in a six-well plate at an initial density of 5 × 10^5^ in 2 mL medium. Overnight cultures were exposed to 34 *μ*g/mL of LE for 24, 48, and 72 hours, respectively. Two percent methanol exposed cells served as a control. Control and experimental groups were set up in duplicate.

### 2.2. Isolation and Quality Control of Total RNA

Total RNA was prepared from control and LE treated MiaPaCa-2 cells using TriReagent (Sigma, USA). At the end of the treatment, cells were directly lysed in TriReagent and total RNA isolated. From this point, the rest of the gene expression related procedures were performed at the microarray and gene expression Core facility of the University of Miami. The quality of the extracted RNA was evaluated using BioAnalyzer 2100 (Agilent Technologies, CA). RNA samples with RNA integrity number (RIN) ≥9 have been used for the microarray experiments [9].

### 2.3. cDNA Synthesis and RNA Labeling for Microarray Analyses

Target RNA was amplified using Amino Allyl Message Amp kit (Ambion, USA). Amplified RNA from the control group was labeled with Cy-3 and the test group with Cy-5 dye (Amersham, USA). Duplicate microarray hybridization was set up using biological replicates that were labeled by dye-swap; that is, control RNA was labeled with Cy-5 and test RNA with Cy-3. The dye labeled cDNA samples were hybridized to the whole human genome microarray (Cat. no. G4112F; Design ID: 014850, Agilent Technologies, USA). We followed the experimental instructions provided by the kit supplier for RNA amplification, cDNA preparation, dye labeling, and array hybridization.

### 2.4. Microarray Image Analyses and Data Processing

The microarrays were scanned at 5 *μ*m resolution using a GenePix 4000B scanner (Molecular Devices, USA). The scanned images were analyzed with GenePix Pro 6.1, the software package provided by Molecular Devices, USA. Quality control of the images was performed with Acuity 4.0 (Molecular Devices, USA) software package. The following quality criteria have been used: (1) at least 90% of the pixels in the spot had intensity higher than background plus two standard deviations, (2) less than 2% saturated pixels in the spot, (3) signal to noise ratio (defined as ratio of the background subtracted mean pixel intensity to standard deviation of background) was 3 or above for each channel, (4) the spot diameter was between 45 and 70 *μ*m, (5) the regression coefficient of ratios of pixel intensity was 0.6 or above, and (6) feature has opposite change direction at dye-swap. Only features that passed all quality control criteria in all microarrays were analyzed further. To identify significantly expressed genes, the R package “limma” was used. “Within array” normalization was carried out with Lowess method, and “between arrays” normalization with “quantile” algorithm in the “limma” package. Features that did not pass the quality control criteria in all arrays were given weight of 0 and were excluded from the analyses. Differential expression and false discovery rate (FDR) were assessed using a linear model and empirical Bayes moderated F statistics. Genes that passed FDR ≤ 0.01 in all time points with a fold change of 2 or more in at least one time point and in the same direction in all time points were uploaded into MetaCore tool (Thomson Reuters, NY) for the identification of pathways affected by LE. The same data sets were uploaded to TM4, microarray data management, and analysis software to generate the heat map [4].

### 2.5. Verification of Microarray Data

The microarray gene expression data were verified by quantitative RT-PCR. Premade validated RT-PCR primers (Table 1) used for data validation were purchased from Real Time Primers, USA. Custom made primers were synthesized by Invitrogen, USA. Real-time RT-PCR was performed with gene-specific primers using the QuantiTect SYBR Green PCR kit (Qiagen, USA) on an Applied Biosystem 5700 RT PCR machine. The PCR conditions were as follows: 10 min at 95°C, 35 cycles at 94°C for 15 seconds, and 30 seconds each at 60°C and 72°C, respectively. The measured transcript abundance was normalized to the level of GAPDH or *β*-actin. The mean normalized expression (MNE) was calculated using the method developed by Muller et al. [10]. The fold change (FC) in gene expression was calculated using the formula FC = MNE of the LE treated/MNE of the control.

## 3. Results

The whole genome microarray (Agilent, USA) featured approximately 41,000, 60 nucleotide long oligonucleotide probes. These probes represent all known human transcripts. The microarray data were deposited in NCBI's “Gene Expression Omnibus” database repository; accession number is GSE44290 [11].

Seven genes differentially regulated in microarray were selected for validation using quantitative real time PCR. The direction of fold change in gene expression observed in microarray analyses matched with the real time PCR data (Table 1).

### 3.1. Primary Screening of Microarray Data Yielded 17,135 Genes That Passed Technical QC in All Microarrays and Have Been Analyzed Further

17,135 features including replicated spots passed technical QC (see “Section 2.4”) in all microarrays. Hybridization signals that passed all the quality control criteria as mentioned in the materials and methods were compared to the signals obtained by dye-swap hybridization. We combined biological replicates for dye swap in each treatment time point. By this method of data filtering, we selected 2,724, 2,730, and 5,463 genes for 24, 48, and 72 h, respectively (see Supplementary File S-1 in Supplementary Materials available online at http://dx.doi.org/10.1155/2013/471739). By this screening, we found that 1178 genes were common for all the three time points irrespective of the direction of expression change viz up or down regulation. Details of these common genes are listed in the Supplementary File S-1. In this data table, the same gene may be upregulated at one time point and downregulated at other time points. These 1,178 genes represented expression data that passed initial QC for all the three time points irrespective of direction of gene expression change triggered by LE treatment. Except for the QC described above, no preset fold change (FC) or false discovery rate (FDR) parameters were applied to these data sets. The lists of genes thus identified to be common for all three time points are detailed in “Spot test” sheet in the Supplementary File S-1.

### 3.2. Application of FDR ≤ 0.01 to Data Set Identified 221 Genes

**Figure 1:**
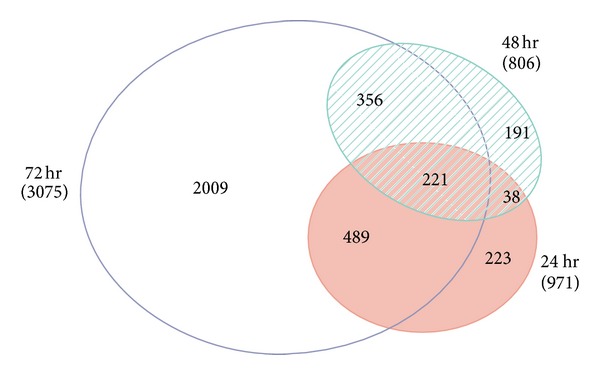
Venn diagram depicting the number of genes regulated by LE at different time points and the number of genes that are common between the time segments (FDR ≤ 0.01). 223, 191, and 2009 genes were unique to 24, 48, and 72 hours, respectively. 259 (221+38) genes were common between 24 and 48 h; 356 between 48 and 72 h; 489 between 24 and 72 h. 221 genes were common among all the three time points. Except for FDR ≤ 0.01, neither fold change nor the direction of regulation is accounted in this representation.

From this data set, we selected genes that passed FDR ≤ 0.01 at each of the three treatment time points. This analyses yielded 971 (637/334), 806 (325/481), and 3,075 (1,758/1,317) genes that were unique to 24 h, 48 h, and 72 h time points, respectively (Supplementary File S-1). The numbers in parenthesis indicate the number of underexpressed and over expressed genes, respectively. A detailed gene list corresponding to the three time points is listed in sheets 24hFDR001, 48hFDR001, and 72hFDR001, respectively (Supplementary File S-1). The genes selected by this method were compared for commonly regulated genes between the different treatment time points. This analysis found 223, 191, and 2,009 genes to be unique to 24, 48, and 72 h, respectively. 38 genes were established to be common between 24 and 48 h, 489 between 24 and 72 hours, and 356 genes between 72 and 48 h. 221 genes were common for all the three time points. Though the genes may be common for the indicated parameters, the direction of regulation may not be the same for these genes at the compared time points. In this list, only the gene names were common. Other than FDR ≤ 0.01 the FC was not factored in this selection. Therefore, the same gene may have a positive and negative fold change at different time points (Sheet “FDR ≤ 0.01,” Supplementary File S-1). This data is graphically represented in Figure 1.

### 3.3. Application of Dual Filter to the Data Set Identified 82 Genes to Be Differentially Regulated by LE

**Figure 2:**
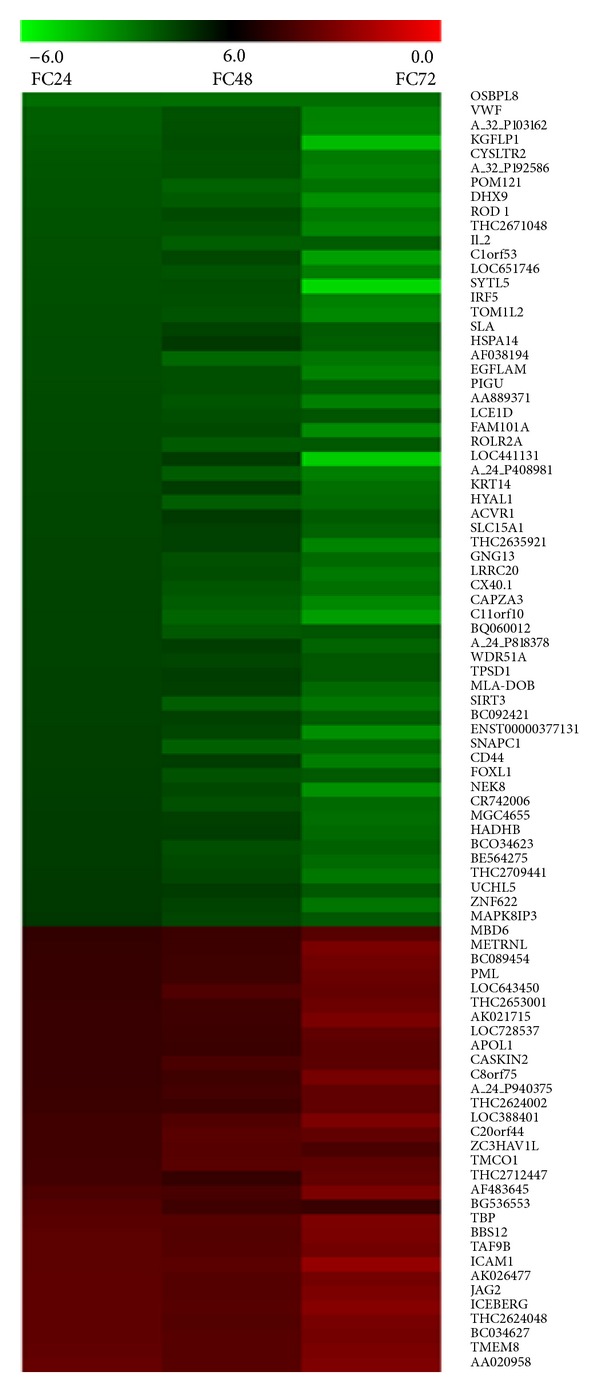
Heat map depicting the relatedness of gene expression pattern in MIAPaCa-2 cells treated with LE. This image was generated using the fold changes in the expression of 89 genes that are significantly down (58; green) or up (31; red) regulated at all the three time points. The intensity of the color is proportional to the fold change in gene expression level between untreated and LE treated MiaPaCa-2 cells. The heat map was generated using TM4, microarray data management, and analysis software [4].

Next, we limited the data analyses to genes that showed similar expression trend either up or down regulated over the three different experimental time points. This analysis yielded the following results. From across three different time points, 941 (516/425) out of 1,178 genes showed similar expression pattern (Sheet “FC Same trend,” Supplementary File S-1). From this list, 362 (248/114) genes showed FC ≥ ±2 (Sheet “FC ≥ ±2,” Supplementary File S-1). This list was further screened for genes with FDR ≤ 0.01 and thus we picked 89 (58/31) genes (Sheet “FDR ≤ 0.01 and FC ≥ ±2,” Supplementary File S-1). Of the 89 genes, 82 had known valid accession IDs. Thus, from 41,000 probe sets on Human Genome microarray, we successfully selected 82 (54/28) genes that showed similar trend across all the three time points with FDR 0.01. In this list, all the genes in at least one of the three points qualified FDR ≤ 0.01 and FC ≥ ±2 (Tables 2(a) and 2(b)). The relatedness of the expression of 89 genes that are significantly down (58) or up (31) regulated at all the three time points is depicted in the heat map (Figure 2).

### 3.4. LE Regulates Key Pathways Involved in Immune Response, Development, and So Forth

**Figure 3:**
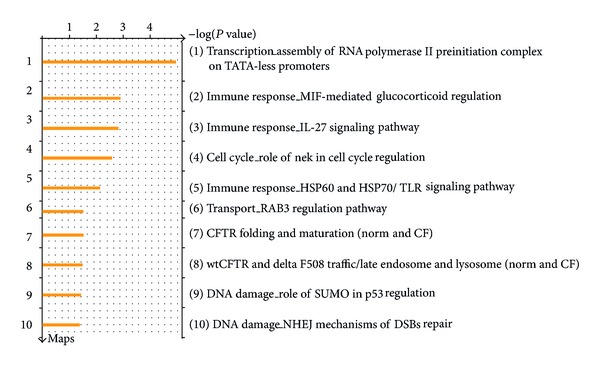
Top ten pathway maps identified by MetaCore tool in the microarray data set of MiaPaCa-2 cells treated with LE for three different time points. The list is arranged per descending *P* value score. A detailed list of all the pathways affected by LE is given in Table 3.

The 82 genes thus selected were uploaded to MetaCore tool in GeneGo suite (GeneGo, USA) to determine the possible signaling pathways affected by LE. This enrichment analysis identified 98 pathways to be regulated by LE. We grouped these 98 pathways that have similar effect. The top ten pathway maps of the 98 identified as above are given in Figure 3. A detailed list of all the 98 pathways affected by LE is given in Table 3. Overall, the data suggested LE predominantly affected immune response (#27), followed by metabolism (#17), developmental (#12), gene expression regulation (#6), cell adhesion (#5), cystic fibrosis transmembrane conductance regulation (CFTR), and chemotaxis (#4) pathways.

**Figure 4:**
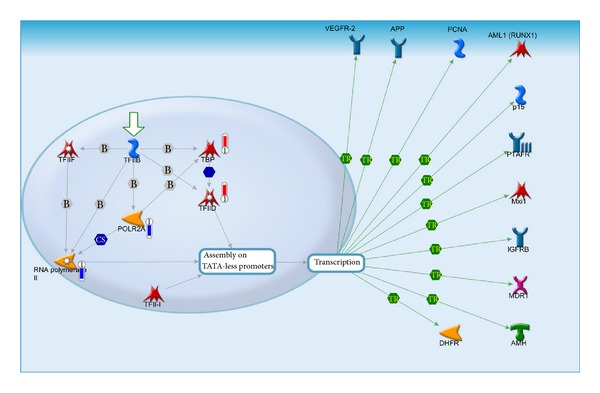
Canonical pathway analyses in MetaCore tool identified Transcription assembly of RNA polymerase II preinitiation complex on TATA-less promoters to be the top scored pathway map (*P* ≤ 1.204*E* − 05) in LE treated cells. Red thermometers show an object that is upregulated by LE. Blue thermometer show the objects downregulated by LE. The exact fold change obtained from three time points with the corresponding SD is given in Table 2. The big arrow indicates the “pathway start.” TR: transcriptional regulation; CS: complex subunit; B: binding; grey arrow: technical link; green arrows: positive effect blue arrows represent positive, red negative, and grey unspecified interactions. Boxes on lines denote the type of regulation where P is phosphorylation, B is binding, and TR is transcriptional regulation. A detailed legend for the objects in this figure is given in the Supplementary File SF-1.

In this analysis, the transcription assembly of RNA Polymerase II preinitiation complex on TATA-less promoters pathway passed QC set at FDR ≤ 0.05. The *P* value was 1.216 × 10^−5^ (Figure 4). Three objects from the uploaded data were found common among the 18 genes that are listed to be involved in this regulation. The key genes involved in this pathway are RNA pol II that is important for the transcription of TATA less promoters. This gene was underexpressed in LE treated cells. It suggests the deregulation of TATA less promoter genes. In genome wide analyses, only 15% of the human genes have been reported to be regulated by classical TATA box containing promoter. Redundancy/overlap of these regulations has been observed in several genes. Therefore, even though it appears that the regulation of TATA less gene transcription is altered, complete loss of their activity may be limited. Otherwise, LE treatment must result in unacceptably high toxicity, which was not encountered in animal studies [7]. Figure 4 illustrates the TF-II and the status of the three genes that are directly involved in this pathway.

Besides the above, additional 11 pathways were significantly affected by LE. They are immune response_MIF-mediated glucocorticoid regulation (*P* ≤ 1.437*E* − 03), immune response_IL-27 signaling (*P* ≤ 1.712*E* − 03), immune response_HSP60 and HSP70/TLR signaling (*P* ≤ 8.466*E* − 03), translation_IL-2 regulation of translation (*P* ≤ 5.066*E* − 02), CFTR folding and maturation (norm and CF) (*P* ≤ 3.572*E* − 02), wtCFTR and delta F508 traffic/Late endosome and lysosome (norm and CF) (*P* ≤ 3.823*E* − 02), protein folding_membrane trafficking and signal transduction of G-alpha (i) heterotrimeric G-protein (*P* ≤ 4.819*E* − 02), DNA damage_role of SUMO in p53 regulation (*P* ≤ 4.322*E* − 02), DNA damage_NHEJ mechanisms of DSBs repair (*P* ≤ 4.819*E* − 02), transport_RAB3 regulation pathway (*P* ≤ 3.572*E* − 02), and cell cycle_role of Nek in cell cycle regulation (*P* ≤ 3.037*E* − 03). The rest of the 86 pathways possibly affected by LE had a *P* value ≥0.05.

### 3.5. Disease Biomarker Analysis Suggests LE Affects Vasculitis

The expression of 82 genes that were identified to be influenced by LE was subjected to disease biomarker enrichment analysis in MetaCore tool. This investigation picked up a large number of diseases from this data set. The top 10 diseases along with the corresponding *P*-values are listed in Table 4. It also contains the number of genes found in the microarray data that matched genes cataloged in MetaCore disease biomarker enrichment database. It appears that LE predominantly affected genes/pathways involved in the inflammation of blood vessels, a condition called Vasculitis. Autoimmune disorders like arthritis and pancreatitis are attributed to be two of many etiology for vasculitis [12, 13]. In this table, arthritis is the second most disease affected by LE (*P* ≤ 6.185*E* − 12). As reported in the literature, *A. aspera* is used as a cure for pancreatic cancer [7], as an antiarthritic medication [14], and to stop bleeding. The outcome of disease network analysis supports its medicinal use. It is notable that LE decreased the CD44 expression (Table 2(a)). The cell surface glycoprotein CD44 is involved in a wide variety of interactions that include receptors, cell-cell interaction, adhesion, and migration (metastasis). Along with CD44, MMPs, VEGF, and hyaluronoglucosaminidase 1 (HYAL1; Table 2(a)), levels decreased in MiaPaCa-2 cells treated with LE. Of these molecules, the inhibition of HYAL1 enzyme was shown to decrease prostate cancer progression *in vivo* [15]. Therefore, LE is likely to suppresses tumor progression and metastasis as demonstrated earlier in our animal studies [7].

## 4. Discussion

In complementary and alternate medicine (CAM), unrefined (crude) preparations of natural products are used for the treatment of various illnesses. It is believed that compounds present in a crude preparation modulate the effect of the active molecule [16]. In this approach, the use of crude extract by CAM practitioners is akin to multidrug therapy. The difference is, in multidrug therapy the chemical nature as well the mechanisms of action of the component drugs are reasonably well studied. Even when combination chemotherapy is practiced, the added drugs are effective only in a few drug combinations [17]. Most of the modern day wonder drugs originated from or derivatives of the active molecules identified from natural products [18, 19]. Once isolated, the active components from these natural products either end up being intolerably toxic, lose their activity, or needed in impractically large doses. Also, the drug discovery pathway is long, arduous, and expensive [20]. While it is ideal to identify the active molecule, we should note that a majority of the world population depends on natural remedies. Shortcomings in the use of natural products as medicine are the lack of quality control of these preparations, dosage optimization and contra indications. Various nodal agencies have embarked on an effort to validate the medicinal benefits, quality control, and dosage standardization of natural products that are widely used by CAM practitioners [1].

We are interested in the anticancer properties of *A. aspera*. It is noteworthy that LE modulates the expression of genes involved in embryonic development, cell adhesion, transcription factors that regulate cell growth, and so forth. These effects support its' use as abortifacient [21]. Hitherto, we have systematically demonstrated that *A. aspera*, a medicinal plant used in Ayurvedic medicine, inhibited the growth of cancer cells *in vitro* and orthotopic pancreatic tumor *in vivo*. Many parts of this plant are used as a cure for a wide array of ailments that include fertility control, fetal expulsion, wound healing, and cancer therapy. Since LE is a crude extract we expected a large number of genes would be affected in the treated cells. However, stringent data filtering determined just 89 genes were affected by LE. Of these, seven were probes used for quality control. Thus, effectively, we have narrowed down the number of genes affected by LE to 82. The pathway data analyses demonstrated predominantly immune response is affected by LE, followed by metabolism, development, transcription, and so forth. The effects on developmental, gene expression regulation, and cell adhesion pathways imply that LE directly affects cancer growth and metastasis.

Mutations in cystic fibrosis gene are identified as a risk factor for chronic pancreatitis and pancreatic ductal adenocarcinoma [22, 23]. Molecular mechanistic study suggest loss of cystic fibrosis transmembrane conductance regulation (CFTR) and a corresponding increase in MUC4 expression is observed in about 81% of pancreatic ductal adenocarcinoma cell lines [24, 25]. The microarray data suggest genes involved in CFTR regulation are modulated by LE. Though the effect of LE on CFTR pathway is highlighted in the gene expression studies, the molecular mechanism by which LE regulates CFTR or related gene function is not known. The short listing of CFTR and other top 10 pathways as affected by LE is worth further investigations. The microarray data are consistent with our experimental findings that LE is preferentially cytotoxic to pancreatic cancer cell lines *in vitro* [8] and the use of LE in the treatment of pancreatic cancer by ayurvedic physicians.

Our results demonstrate that it is possible to pin point possible mechanism of action of crude extract using carefully planned experiments. We applied several assumptions in our data analyses. For instance, we eliminated expressions that did not follow the same pattern. There are instances where a group of genes may be up regulated in the short term and down regulated over long term exposure to LE or vice versa. Though less likely, another set of genes may be up and down regulated in a cyclical manner. By considering unidirectional regulation, we would have eliminated such unique changes effected by LE. Ambiguities in gene expression analysis may be reduced and the robustness of expression data may be increased by using several experimental replicates, multiple different cell lines, and different drug concentrations. Additionally, gene expression analyses may be performed in tumor samples obtained from animal model that had been treated with LE.

The identification of regulation of 82 genes from a large data set encourages us to think that natural products may target a limited set of genes. There are evidences to support such observations. For instance, almost identical results were obtained in MCF-7 breast cancer cells that were treated with a crude extract of *Anoectochilus formosanus* and a single compound drug plumbagin [26]. However, natural products may have much broader impact on global gene expression pattern. Therefore, we must be cautious in the universality of this approach.

In conclusion, we have demonstrated that LE affected the expression of only a few genes. Using similar methodology, it is possible to get reasonably good overview of the changes induced by crude preparation of natural products with medicinal properties.

## Supplementary Material

The gene expression data is deposited with the NCBI's “Gene Expression Omnibus” database repository under the accession number GSE44290.
Supplementary data Table S-1 and supplementary Figure SF-1 are available online at http://dx.doi.org/10.1155/2013/471739. The data referred in sections 3.1, 3.2 & 3.3 are included in the supplementary data Table S-1. The legends for the objects in Figure 4 are given in the supplementary Figure SF-1.

## Figures and Tables

**Table 1:** Eight genes were analyzed to validate the microarray data. The fold changes in gene expression as obtained by microarray analyses were verified by quantitative RT-PCR. Experimental details are given in the materials and methods.

No.	Gene symbol	Gene name	Microarray FC	RT PCR FC
1	TBP	TATA box binding protein	2.37	1.29
2	APOL1	Apolipoprotein L	1.64	3.59
3	ICAM1	Intercellular adhesion molecule 1	2.63	1.94
4	POLR2A	Polymerase (RNA) II (DNA directed) polypeptide A	−1.99	0.69
5	SLA	Src-like-adaptor	−1.85	0.43
6	HYAL1	Hyaluronoglucosaminidase 1	−2.12	0.42
7	IRF5	Interferon regulatory factor 5	−2.25	0.89
8	IL2	Interleukin 2	−2.10	0.58

**Table:** (a)

No.	Gene name	NCBI description	AVE FC	SD
1	SYTL5	Synaptotagmin-like 5	−2.92	1.88
2	KGFLP1	Fibroblast growth factor 7 pseudogene	−2.80	1.42
3	ARPC3P5	Actin related protein 2/3 complex, subunit 3 pseudogene 5	−2.63	1.88
4	OSBPL8	Oxysterol binding protein-like 8	−2.62	0.01
5	C11orf10	Chromosome 11 open reading frame 10	−2.56	1.09
6	DHX9	DEAH (Asp-Glu-Ala-His) box polypeptide 9	−2.51	0.77
7	C1orf53	Chromosome 1 open reading frame 53	−2.45	1.16
8	VWF	Von Willebrand factor	−2.40	0.59
9	AF038194	Homo sapiens clone 23821 mRNA sequence	−2.35	0.51
10	POM121	POM121 transmembrane nucleoporin	−2.33	0.36
11	THC2671048	Q3DWD9_CHLAU (Q3DWD9) YLP motif, partial (6%)	−2.33	0.71
12	TOM1L2	Target of myb1-like 2 (chicken)	−2.33	0.76
13	CAPZA3	Capping protein (actin filament) muscle Z-line, alpha 3	−2.33	0.82
14	CYSLTR2	Cysteinyl leukotriene receptor 2	−2.30	0.54
15	ANKRD33B	Ankyrin repeat domain 33B	−2.27	0.61
16	AA889371	am40h08.s1 Soares_NFL_T_GBC_S1 Homo sapiens cDNA clone IMAGE:1471263 3′ similar to SW:COQ1_YEAST P18900 HEXAPRENYL PYROPHOSPHATE SYNTHETASE	−2.26	0.67
17	FAM101A	Family with sequence similarity 101, member A	−2.26	0.91
18	IRF5	Interferon regulatory factor 5	−2.25	0.68
19	EGFLAM	EGF-like, fibronectin type III, and laminin G domains	−2.25	0.72
20	SIRT3	NAD-dependent protein deacetylase sirtuin-3, mitochondrial isoform a	−2.20	0.65
21	NEK8	NIMA (never in mitosis gene a)—related kinase 8	−2.19	1.07
22	ROD1	Polypyrimidine tract binding protein 3	−2.18	0.60
23	RBM26	RNA binding motif protein 26	−2.18	1.05
24	HYAL1	Hyaluronoglucosaminidase 1 (HYAL1), transcript variant 1, noncoding RNA	−2.12	0.45
25	IL2	Interleukin 2	−2.10	0.17
26	LRRC20	Leucine-rich repeat-containing protein 20 isoform 3	−2.10	0.67
27	THC2635921		−2.09	0.90
28	GJD4	Gap junction protein, delta 4, 40.1 kDa	−2.09	0.52
29	SNAPC1	Small nuclear RNA activating complex, polypeptide 1, 43 kDa	−2.06	0.50
30	GNG13	Guanine nucleotide binding protein (G protein), gamma 13	−2.00	0.45
31	POLR2A	Polymerase (RNA) II (DNA directed) polypeptide A, 220 kDa	−1.99	0.23
32	CD44	CD44 molecule (Indian blood group)	−1.97	0.87
33	CR742006	CR742006 Soares_testis_NHT Homo sapiens cDNA clone IMAGp971J1256; IMAGE:1048737 5′, mRNA sequence	−1.96	0.53
34	THC2709441	Q4VIX2_DROBU (Q4VIX2) Dbuz*∖*abd-A-PB, partial (4%)	−1.95	0.77
35	PIGU	Phosphatidylinositol glycan anchor biosynthesis, class U	−1.95	0.25
36	KRT14	Keratin 14	−1.91	0.62
37	BE564275	601343077F1 NIH_MGC_53 Homo sapiens cDNA clone IMAGE:3685338 5′, mRNA sequence	−1.90	0.59
38	ZNF622	Zinc finger protein 622	−1.89	0.76
39	BQ060012	AGENCOURT_6793913 NIH_MGC_99 Homo sapiens cDNA clone IMAGE:5816175 5′, mRNA sequence	−1.88	0.28
40	LCE1D	Late cornified envelope 1D	−1.88	0.13
41	BC034623	Homo sapiens cDNA clone IMAGE:4837603	−1.86	0.45
42	SLA	Src-like-adaptor	−1.85	0.30
43	HLA-DOB	Major histocompatibility complex, class II, DO beta	−1.85	0.57
44	FOXL1	Forkhead box L1	−1.84	0.34
45	B3GNT9	UDP-GlcNAc:betaGal beta-1, 3-N-acetylglucosaminyltransferase 9	−1.84	0.66
46	SLC15A1	Solute carrier family 15 (oligopeptide transporter), member 1	−1.82	0.43
47	HADHB	hydroxyacyl-CoA dehydrogenase/3-ketoacyl-CoA thiolase/enoyl-CoA hydratase (trifunctional protein), beta subunit	−1.79	0.61
48	HSPA14	Heat shock 70 kDa protein 14	−1.78	0.43
49	BC092421	Homo sapiens cDNA clone IMAGE:30378758	−1.76	0.39
50	POC1A	POC1 centriolar protein homolog A (Chlamydomonas)	−1.75	0.27
51	ACVR1	Activin A receptor, type I	−1.72	0.41
52	TPSD1	Tryptase delta 1	−1.68	0.33
53	MAPK8IP3	Mitogen-activated protein kinase 8 interacting protein 3	−1.67	0.40
54	UCHL5	Ubiquitin carboxyl-terminal hydrolase L5	−1.61	0.41

**Table:** (b)

No.	Gene name	NCBI description	AVE FC	SD
1	MBD6	Methyl-CpG binding domain protein 6	1.58	0.44
2	BG536553	602564961F1 NIH_MGC_77 Homo sapiens cDNA clone IMAGE:4689518 5′, mRNA sequence	1.64	0.34
3	APOL1	Apolipoprotein L, 1	1.64	0.44
4	LOC728537	Uncharacterized LOC728537	1.69	0.52
5	THC2624002	Q9BXR7_HUMAN (Q9BXR7) Interleukin 10 (Fragment), partial (93%)	1.72	0.49
6	CASKIN2	CASK interacting protein 2	1.77	0.41
7	PML	Promyelocytic leukemia	1.78	0.66
8	ZC3HAV1L	Zinc finger CCCH-type, antiviral 1-like	1.81	0.25
9	THC2653001	BX098637 Soares fetal liver spleen 1NFLS Homo sapiens cDNA clone IMAGp998F16386; IMAGE:200847, mRNA sequence	1.81	0.67
10	LOC728344	Glutaredoxin 3 pseudogene	1.88	0.56
11	METRNL	Meteorin, glial cell differentiation regulator-like	1.89	0.89
12	C8orf75	Long intergenic nonprotein coding RNA 589	1.91	0.81
13	AK021715	Homo sapiens cDNA FLJ11653 fis, clone HEMBA1004538	1.91	0.86
14	TMCO1	Transmembrane and coiled-coil domains 1	1.99	0.36
15	UQCC	Ubiquinol-cytochrome c reductase complex chaperone	2.00	0.41
16	RPL7P48	Ribosomal protein L7 pseudogene 48	2.14	0.73
17	AF483645	Homo sapiens capacitative calcium channel protein Trp1 mRNA, partial cds; alternatively spliced	2.17	0.67
18	TAF9B	TAF9B RNA polymerase II, TATA box binding protein (TBP)-associated factor, 31 kDa	2.31	0.37
19	AK026477	Homo sapiens cDNA:FLJ22824 fis, clone KAIA3991	2.34	0.39
20	BBS12	Bardet-Biedl syndrome 12	2.37	0.48
21	THC2624048	Q964F8_PLAFA (Q964F8) Merozoite surface protein 8, partial (3%)	2.37	0.44
22	TBP	TATA box binding protein	2.37	0.50
23	BC034627	Homo sapiens cDNA clone IMAGE:4839213	2.38	0.36
24	JAG2	Jagged 2	2.42	0.49
25	TMEM8	Transmembrane protein 8A	2.42	0.43
26	CARD18	Caspase recruitment domain family, member 18	2.51	0.59
27	AA020958	ze65a02.s1 Soares retina N2b4HR Homo sapiens cDNA clone IMAGE:363818 3′, mRNA sequence	2.52	0.48
28	ICAM1	Intercellular adhesion molecule 1	2.63	0.77

**Table 3:** Signaling pathways affected by LE in Pancreatic cancer cells. The experimental data of 82 genes (see “Section 3.3”) were uploaded to Metacore tool. It identified 98 pathways to be regulated by LE. The number of pathways affected under each category is indicated in parenthesis.

No.	Pathway map	*P* value	Ratio
	**Immune response** (27)		
1	Immune response_MIF-mediated glucocorticoid regulation	1.437*E* − 03	2 : 22
2	Immune response_IL-27 signaling pathway	1.712*E* − 03	2 : 24
3	Immune response_HSP60 and HSP70/TLR signaling pathway	8.466*E* − 03	2 : 54
4	Immune response_IFN alpha/beta signaling pathway	6.049*E* − 02	1 : 24
5	Immune response_role of HMGB1 in dendritic cell maturation and migration	6.780*E* − 02	1 : 27
6	Immune response_CD137 signaling in immune cell	7.265*E* − 02	1 : 29
7	Immune response_Delta-type opioid receptor signaling in T-cells	7.265*E* − 02	1 : 29
8	Immune response_IL-22 signaling pathway	8.465*E* − 02	1 : 34
9	CCR4-dependent immune cell chemotaxis in asthma and atopic dermatitis	8.465*E* − 02	1 : 34
10	Mechanism of action of CCR4 antagonists in asthma and atopic dermatitis (Variant 1)	8.465*E* − 02	1 : 34
11	Immune response_regulation of T cell function by CTLA-4	8.941*E* − 02	1 : 36
12	Immune response_role of integrins in NK cells cytotoxicity	9.415*E* − 02	1 : 38
13	Immune response_Th1 and Th2 cell differentiation	9.886*E* − 02	1 : 40
14	Immune response_IL-5 signalling	1.082*E* − 01	1 : 44
15	Immune response_PGE2 signaling in immune response	1.105*E* − 01	1 : 45
16	Immune response_NF-AT signaling and leukocyte interactions	1.129*E* − 01	1 : 46
17	Immune response_histamine H1 receptor signaling in immune response	1.175*E* − 01	1 : 48
18	Immune response_IL-2 activation and signaling pathway	1.198*E* − 01	1 : 49
19	Immune response_function of MEF2 in T lymphocytes	1.221*E* − 01	1 : 50
20	Immune response_HMGB1/RAGE signaling pathway	1.289*E* − 01	1 : 53
21	Immune response _IFN gamma signaling pathway	1.312*E* − 01	1 : 54
22	Immune response_CCR5 signaling in macrophages and T lymphocytes	1.402*E* − 01	1 : 58
23	Immune response_immunological synapse formation	1.425*E* − 01	1 : 59
24	Immune response_TREM1 signaling pathway	1.425*E* − 01	1 : 59
25	Immune response_IL-17 signaling pathways	1.447*E* − 01	1 : 60
26	Immune response_CD40 signaling	1.558*E* − 01	1 : 65
27	Immune response_CD16 signaling in NK cells	1.646*E* − 01	1 : 69

	**Metabolism **(17)		
28	Mitochondrial ketone bodies biosynthesis and metabolism	6.780*E* − 02	1 : 27
29	Propionate metabolism p.1	9.651*E* − 02	1 : 39
30	Selenoamino acid metabolism	1.312*E* − 01	1 : 54
31	Phenylalanine metabolism/rodent version	1.580*E* − 01	1 : 66
32	Propionate metabolism p.2	1.580*E* − 01	1 : 66
33	Phenylalanine metabolism	1.602*E* − 01	1 : 67
34	Leucine, isoleucine and valine metabolism.p.2	1.841*E* − 01	1 : 78
35	Leucine, isoleucine, and valine metabolism/Rodent version	1.884*E* − 01	1 : 80
36	Tyrosine metabolism p.2 (melanin)	1.947*E* − 01	1 : 83
37	Lysine metabolism	1.968*E* − 01	1 : 84
38	Lysine metabolism/rodent version	2.010*E* − 01	1 : 86
39	GTP-XTP metabolism	2.094*E* − 01	1 : 90
40	Tryptophan metabolism	2.319*E* − 01	1 : 101
41	Tryptophan metabolism/rodent version	2.339*E* − 01	1 : 102
42	CTP/UTP metabolism	2.459*E* − 01	1 : 108
43	NAD metabolism	2.674*E* − 01	1 : 119
44	ATP/ITP metabolism	2.770*E* − 01	1 : 124

	**Development** (12)		
45	Development_glucocorticoid receptor signaling	6.049*E* − 02	1 : 24
46	Development_cross-talk between VEGF and angiopoietin 1 signaling pathways	6.537*E* − 02	1 : 26
47	Development_osteopontin signaling in osteoclasts	7.506*E* − 02	1 : 30
48	Development_BMP signaling	8.226*E* − 02	1 : 33
49	Development_lipoxin inhibitory action on PDGF, EGF, and LTD4 signaling	8.941*E* − 02	1 : 36
50	Development_beta-adrenergic receptors transactivation of EGFR	9.179*E* − 02	1 : 37
51	Development_notch signaling pathway	1.059*E* − 01	1 : 43
52	Development_S1P3 receptor signaling pathway	1.059*E* − 01	1 : 43
53	Development_VEGF signaling and activation	1.059*E* − 01	1 : 43
54	Development_S1P1 signaling pathway	1.082*E* − 01	1 : 44
55	Development_beta-adrenergic receptors regulation of ERK	1.152*E* − 01	1 : 47
56	Development_WNT signaling pathway, part 2	1.289*E* − 01	1 : 53

	**Gene expression regulation** (6)		
57	Transcription_assembly of RNA polymerase II preinitiation complex on TATA-less promoters	1.204*E* − 05	3 : 18
58	Translation_IL-2 regulation of translation	5.066*E* − 02	1 : 20
59	Transcription_role of akt in hypoxia induced HIF1 activation	6.780*E* − 02	1 : 27
60	Transcription_ligand-dependent transcription of retinoid-target genes	8.226*E* − 02	1 : 33
61	Transcription_role of AP-1 in regulation of cellular metabolism	9.415*E* − 02	1 : 38
62	Translation_(L)-selenoaminoacids incorporation in proteins during translation	1.012*E* − 01	1 : 41

	**Cell adhesion** (5)		
63	Cell adhesion_IL-8-dependent cell migration and adhesion	8.226*E* − 02	1 : 33
64	Cell adhesion_cell-matrix glycoconjugates	9.415*E* − 02	1 : 38
65	Cell adhesion_ephrin signaling	1.105*E* − 01	1 : 45
66	Cell adhesion_ECM remodeling	1.267*E* − 01	1 : 52
67	Cell adhesion_chemokines and adhesion	2.299*E* − 01	1 : 100

	**CFTR regulation** (4)		
68	CFTR folding and maturation (norm and CF)	3.572*E* − 02	1 : 14
69	wtCFTR and delta F508 traffic/late endosome and lysosome (norm and CF)	3.823*E* − 02	1 : 15
70	Regulation of degradation of delta F508 CFTR in CF	6.780*E* − 02	1 : 27
71	Mechanisms of CFTR activation by S-nitrosoglutathione (normal and CF)	1.129*E* − 01	1 : 46

	**Chemotaxis** (4)		
72	Chemotaxis_CCR4-induced chemotaxis of immune cells	8.465*E* − 02	1 : 34
73	Chemotaxis_lipoxin inhibitory action on fMLP-induced neutrophil chemotaxis	1.129*E* − 01	1 : 46
74	Chemotaxis_inhibitory action of lipoxins on IL-8- and leukotriene B4-induced neutrophil migration	1.244*E* − 01	1 : 51
75	Chemotaxis_leukocyte chemotaxis	1.777*E* − 01	1 : 75

	**Oxidative stress** (4)		
76	Oxidative stress_role of ASK1 under oxidative stress	8.465*E* − 02	1 : 34
77	Mitochondrial unsaturated fatty acid beta-oxidation	1.105*E* − 01	1 : 45
78	Mitochondrial long chain fatty acid beta-oxidation	1.947*E* − 01	1 : 83
79	Peroxisomal branched chain fatty acid oxidation	1.947*E* − 01	1 : 83

	**Protein degradation** (4)		
80	Protein folding_membrane trafficking and signal transduction of G-alpha (i) heterotrimeric G-protein	4.819*E* − 02	1 : 19
81	Proteolysis_putative ubiquitin pathway	5.804*E* − 02	1 : 23
82	Proteolysis_role of parkin in the ubiquitin-proteasomal pathway	6.049*E* − 02	1 : 24
83	Proteolysis_putative SUMO-1 pathway	7.265*E* − 02	1 : 29

	**Blood coagulation** (3)		
84	Blood coagulation_blood coagulation	9.651*E* − 02	1 : 39
85	Blood coagulation_GPVI-dependent platelet activation	1.335*E* − 01	1 : 55
86	Blood coagulation_GPIb-IX-V-dependent platelet activation	1.798*E* − 01	1 : 76

	**DNA damage** (3)		
87	DNA damage_role of SUMO in p53 regulation	4.322*E* − 02	1 : 17
88	DNA damage_NHEJ mechanisms of DSBs repair	4.819*E* − 02	1 : 19
89	DNA damage_nucleotide excision repair	8.941*E* − 02	1 : 36

	**Apoptosis** (2)		
90	Apoptosis and survival_role of IAP-proteins in apoptosis	7.747*E* − 02	1 : 31
91	Apoptosis and survival_lymphotoxin-beta receptor signaling	1.036*E* − 01	1 : 42

	**Cytoskeleton remodeling** (2)		
92	Cytoskeleton remodeling_keratin filaments	8.941*E* − 02	1 : 36
93	Cytokine production by Th17 cells in CF	9.651*E* − 02	1 : 39

	**Other** (3)		
94	Transport_RAB3 regulation pathway	3.572*E* − 02	1 : 14
95	Inhibitory action of lipoxin A4 on PDGF, EGF and LTD4 signaling	8.704*E* − 02	1 : 35
96	Signal transduction_calcium signaling	1.105*E* − 01	1 : 45
97	Inhibitory action of lipoxins on neutrophil migration	1.380*E* − 01	1 : 57

	**Cell cycle** (1)		
98	Cell cycle_role of Nek in cell cycle regulation	3.037*E* − 03	2 : 32

**Table 4:** A partial list of diseases that may be affected by LE. The top ten diseases identified by MetaCore tool in the microarray data set of MiaPaCa-2 cells treated with LE are listed in this table. The list is arranged per descending *P* value. The number of genes in the uploaded microarray data versus the genes cataloged in MetaCore disease biomarker enrichment database is indicated as ratio.

No.	Diseases	*P*-value	Ratio
1	Rheumatoid vasculitis	4.375*E* − 13	6 : 10
2	Arthritis, experimental	6.185*E* − 12	6 : 14
3	Papilloma, intraductal	1.789*E* − 10	4 : 4
4	Systemic vasculitis	3.926*E* − 10	7 : 47
5	DNA virus infections	9.529*E* − 09	15 : 654
6	Herpesviridae infections	1.106*E* − 08	12 : 378
7	Vasculitis	1.573*E* − 08	10 : 238
8	Lymphangiomyoma	3.694*E* − 08	4 : 10
9	Smooth muscle tumor	3.694*E* − 08	4 : 10
10	Lymphangioleiomyomatosis	3.694*E* − 08	4 : 10
