# Supplementary material for: In Vitro Global Gene Expression Analyses Support the Ethnopharmacological Use of Achyranthes aspera
Source: Evid Based Complement Alternat Med. 2013 Dec 15;2013:471739. doi: 10.1155/2013/471739 (PMC3880711; doi:10.1155/2013/471739)
Supplement: Supplementary file 2 [file 471739.f2.pdf]

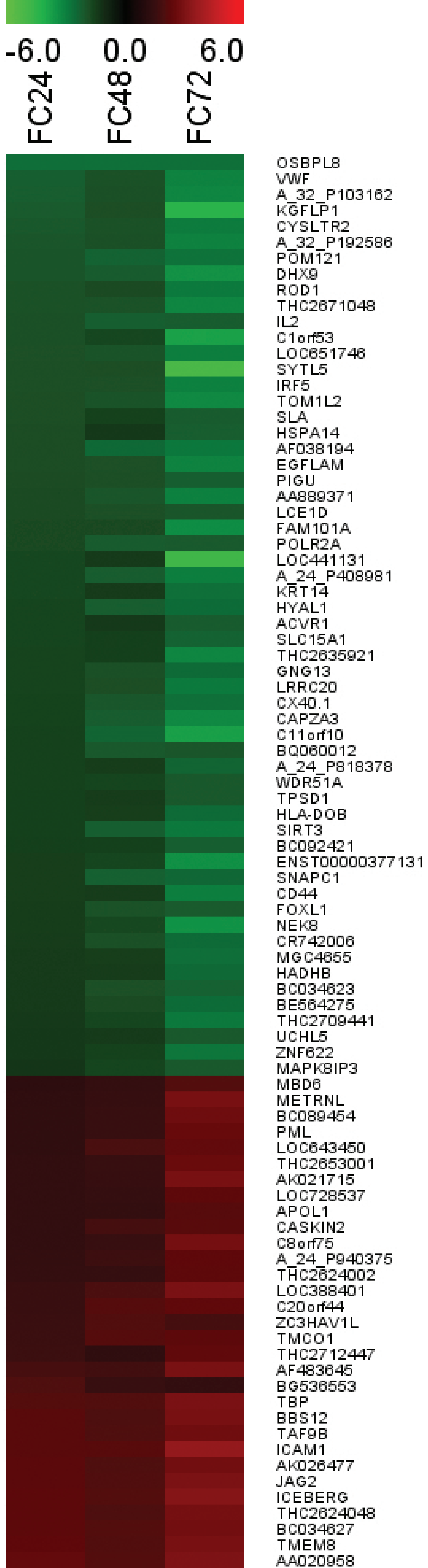
-6.0
0.0
6.0
FC24
FC48
FC72
OSBPL8
VWF
A_32_P103162
KGFLP1
CYSLTR2
A_32_P192586
POM121
DHX9
ROD1
THC2671048
IL2
C1orf53
LOC651746
SYTL5
IRF5
TOM1L2
SLA
HSPA14
AF038194
EGFLAM
PIGU
AA889371
LCE1D
FAM101A
POLR2A
LOC441131
A_24_P408981
KRT14
HYAL1
ACVR1
SLC15A1
THC2635921
GNG13
LRRC20
CX40.1
CAPZA3
C11orf10
BQ060012
A_24_P818378
WDR51A
TPSD1
HLA-DOB
SIRT3
BC092421
ENST00000377131
SNAPC1
CD44
FOXL1
NEK8
CR742006
MGC4655
HADHB
BC034623
BE564275
THC2709441
UCHL5
ZNF622
MAPK8IP3
MBD6
METRNL
BC089454
PML
LOC643450
THC2653001
AK021715
LOC728537
APOL1
CASKIN2
C8orf75
A_24_P940375
THC2624002
LOC388401
C20orf44
ZC3HAV1L
TMCO1
THC2712447
AF483645
BG536553
TBP
BBS12
TAF9B
ICAM1
AK026477
JAG2
ICEBERG
THC2624048
BC034627
TMEM8
AA020958
